# CK17 and p16 expression patterns distinguish (atypical) immature squamous metaplasia from high-grade cervical intraepithelial neoplasia (CIN III)

**DOI:** 10.1111/j.1365-2559.2007.02652.x

**Published:** 2007-04-01

**Authors:** S Regauer, O Reich

**Affiliations:** Institute of Pathology, Medical University Graz Graz, Austria; 1 Department of Obstetrics and Gynaecology, Medical University Graz Graz, Austria

**Keywords:** HPV, reactive atypia, squamous intraepithelial lesions

## Abstract

Regauer S & Reich O (2007) *Histopathology***50,** 629–635 **CK17 and p16 expression patterns distinguish (atypical) immature squamous metaplasia from high-grade cervical intraepithelial neoplasia (CIN III)**

**Aims:**

Atypical immature metaplasia (AIM) refers to a full-thickness intraepithelial basaloid lesion in the uterine cervix that features both metaplasia and atypia and is therefore difficult to distinguish from high-grade cervical intraepithelial neoplasia (CIN III). p16 is a marker for human papillomavirus (HPV)-induced dysplasia. Cytokeratin (CK) 17 is a marker for cervical reserve (stem) cells, which give rise to metaplasia. The aim was to determine whether AIM can be reclassified into metaplasia and CIN III based on p16 and CK17 immunohistochemistry.

**Material and results:**

Seventy-five cervical biopsy specimens, curettings and cone excisions containing varying proportions of dysplasia and metaplasia and 20 cases regarded as AIM were analysed immunohistochemically with antibodies to CK17, p16 and p63. In immature metaplasia all proliferating cells were immunoreactive with antibodies to CK17 and p63, while p16 was negative. All dysplastic cells of CIN III demonstrated uniform immunoreactivity for p16 and p63, but were CK17–. Based on the reciprocal immunoreactivity of p16 and CK17, 17/20 cases of AIM were reclassified as metaplasia (*n* = 10) and CIN III (*n* = 7). Three cases of AIM stained for both CK17 and p16 and were classified as CIN III.

**Conclusion:**

‘AIM’ is a helpful histological descriptor but it should not be used as a final diagnosis. Immunohistochemistry for p16 and CK17 allows distinction between metaplasia and high-grade CIN.

## Introduction

Many squamous intraepithelial lesions (SIL) of the uterine cervix can be classified according to histological criteria into metaplastic and dysplastic processes. A significant number of SIL involving the entire thickness of the epithelium, however, are problematic due to preservation of a metaplastic phenotype. The term atypical immature squamous metaplasia (AIM) was initially introduced in 1983 to describe lesions in the uterine cervix featuring a uniform intraepithelial full-thickness basal cell proliferation with high nuclear density in the absence of maturation but without sufficient criteria for a diagnosis of high-grade cervical intraepithelial neoplasia (CIN III).^1^ In the initial description, AIM was identified together with condyloma accuminatum in 34% and with CIN III in 16%. Since this first description, papillary lesions of AIM have been reclassified as immature condylomas.^2^–^4^ Non-papillary atypical immature squamous proliferations with cytological atypia sufficient for a diagnosis of high-grade dysplasia and some metaplastic features have been recently termed eosinophilic dysplasia.^5^ Other non-papillary atypical immature squamous proliferations which contain both metaplastic features and cytological atypia that defy precise classification were termed several years ago as ‘atypical immature metaplastic-like proliferation of the cervix’.^6^ The diagnosis of AIM has poor intra- and interobserver reproducibility on haematoxylin and eosin (H&E)-stained sections because of its resemblance to CIN III. The biological and clinical significance of a diagnosis of AIM is unclear. Application of adjuvant markers is needed for further subclassification of AIM into metaplasia and high-grade SIL. Immunohistochemical demonstration of p16 overexpression with an antibody to p16 has emerged as a surrogate marker for CIN induced by high-risk human papillomavirus (HPV).^7^–^9^ The oncogenes encoded by the *E6* and *E7* genes of HPV bind to host cell regulatory proteins, especially to tumour suppressor gene products p53 and hypo-phosphorylated retinoblastoma protein (pRb). p16 is a tumour suppressor protein that inhibits cyclin-dependent kinases (CDK)-4 and CDK-6, which regulate the G_1_ checkpoint. The CDKs phosphorylate pRb, which results in a conformational change and release of E2F from pRb. Functional pRb controls p16 transcription via a negative feed-back loop. The Rb tumour suppressor function is functionally inactivated by HPV E7 oncoproteins, which results in p16 overexpression in cervical cancers.^10^, ^11^ Inactivation of pRb function allows the cell to enter the S-phase after only a pause at the G_1_ checkpoint. Viral oncongenes of low-risk HPV have no effect on p16/cyclin D1/cdk4/pRb complexes, because the affinity of low-risk HPV E7 protein for cellular pRb is 10-fold lower than that of HPV-16 E7 for pRb.^12^ Low-grade dysplasia (CIN I) may, occasionally, be p16+. CIN I and to some extent CIN II, however, are not a differential diagnosis for AIM and CIN III on H&E pathology, since CIN I and CIN II show maturation and keratinization of the upper cell layers. Cytokeratin (CK) 17 recognizes cervical stem cells^13^ and it is expressed in immature metaplastic squamous epithelium.^14^ p63, a homologue of the *p53* suppressor gene, is a proliferation marker that is expressed in basal keratinocytes of glycogenated squamous mucosa, in reserve cells of the transformation zone and in immature squamous epithelium, but not in endocervical epithelium. Early squamous (immature) metaplasia therefore is characterized by an expanding population of p63+ and CK17+ subcolumnar cells in the transformation zone.^15^ In this study, we tested the hypothesis that immature metaplasia with or without (reactive) atypia reveals strong cytoplasmic CK17 immunoreactivity of the proliferating cells in the absence of p16 staining, while high-grade dysplastic lesions demonstrate p16 positivity along with CK17 negativity.

## Materials and methods

We investigated a total of 75 formalin-fixed paraffin-embedded cervical specimens: cervical punch biopsies (*n* = 30), knife cone and loop electrosurgical excision procedure (LEEP) excision specimens (*n* = 30) and endocervical curettings (*n* = 15) with varying proportions of mature metaplasia, immature metaplasia, low-grade SIL (= CIN I) and high-grade SIL (= CIN II and CIN III) from the archives of the Institute of Pathology and the Department of Gynaecology, Medical University Graz, Austria and compared the staining pattern to 20 cases signed out as AIM. All cases were analysed immunohistochemically using antibodies to CK17 and p63 (Dako, Glostrup, Denmark) and p16 (clone E6H4, CINtec Histology Kit, mtm Laboratories, Heidelberg, Germany) with microwave retrieval (750 W, 10 min) on a Dako Autostainer K5001 according to standard protocols.

p16 was classified as positive^5^, ^7^ when intense diffuse cytoplasmic and nuclear immunoreactivity of the entire cervical squamous epithelium was observed. Focal scattered cytoplasmic and/or nuclear immunoreactivity was recorded as negative. CK17 immunoreactivity was cytoplasmic and was scored positive as soon as a single cell was stained.

## Results

**Figure 1:**
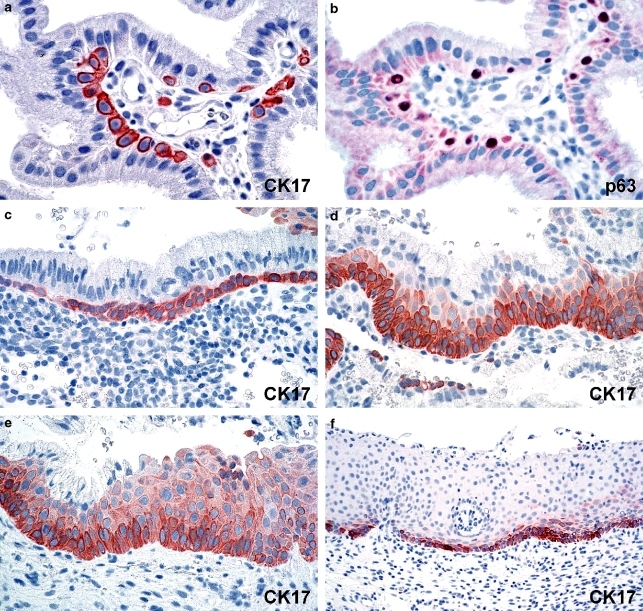
The metaplastic process in the cervix begins with individual reserve cell proliferation: cytoplasmic cytokeratin (CK) 17 staining of subcolumnar cells (**a**) and nuclear p63 staining (**b**) Early immature metaplasia shows linear CK17 staining (**c**) which progresses to full-thickness staining in immature metaplasia (**d,e**) The preserved columnar endocervical cells are negative. As the metaplasia matures it becomes negative for CK19 with occasional retained basal keratinocyte staining (**f**).

In native cervical epithelium, neither ecto- nor endocervical epithelium reacted with the antibody to p16. The columnar endocervical epithelium showed no staining with antibody to CK17, while CK17 was inconsistently, focally and weakly expressed in the cytoplasm of basal keratinocytes in ectocervical glycogenated squamous epithelium. The antibody to p63 reacted with basal keratinocyte nuclei in the glycogenated squamous epithelium, but showed no reaction in the columnar endocervical epithelium. Metaplastic epithelium was divided into mature and immature forms. The earliest phase of immature metaplasia was a proliferation of individual reserve cells. CK17 stained the cytoplasm (Figure 1a) and p63 the nuclei (Figure 1b). The proliferating cells of immature squamous metaplasia were consistently positive for CK17 in the subcolumnar reserve cells (Figure 1c) and in the proliferating basal and suprabasal cells (Figure 1d), while the columnar cells—where preserved above the basal cell proliferation—were negative (Figure 1d,e). Expression of CK17 decreased and disappeared as the metaplastic epithelium matured, although expression did not always disappear completely (Figure 1f). Mature metaplasia was histologically indistinguishable from ectocervical glycogenated mucosa and showed an identical staining profile (an occasional CK17+ basal keratinocyte, linear p63 positivity of basal keratinocyte nuclei). Immature and mature metaplasia was consistently negative for p16.

**Figure 2:**
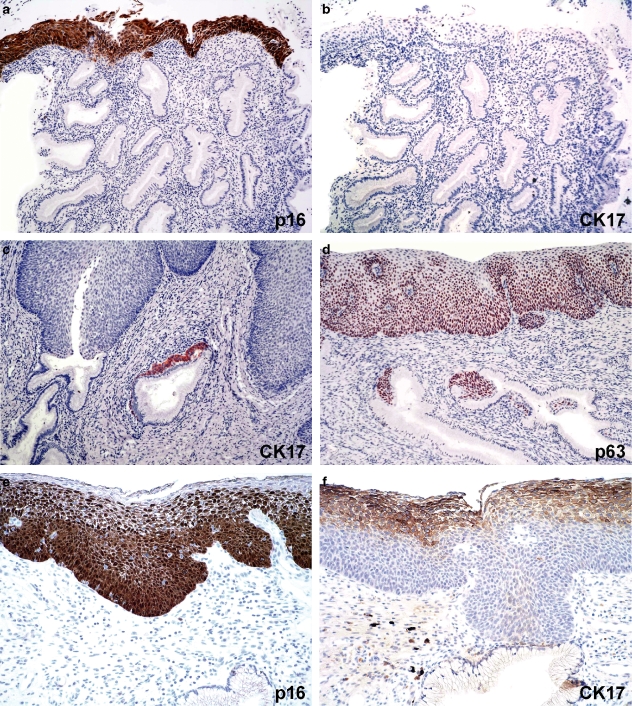

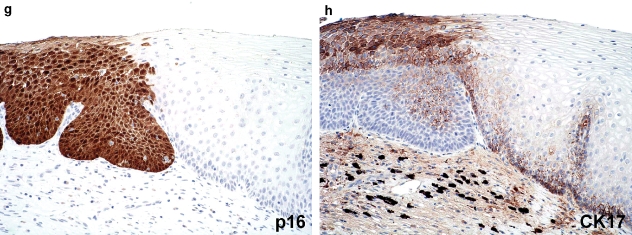
Cervical intraepithelial neoplasia (CIN) III with p16+ (**a**) and cytokeratin (CK) 17– (**b**) surface epithelium. Only metaplastic cells in a gland beneath the dysplastic surface epithelium stain positive for CK17 (**c**) p63 immunoreactive nuclei of the dysplastic epithelium (**d**) In lesions of CIN II, antibody to p16 shows near full-thickness positivity (**e**), while antibody to CK17 reacts only with the metaplastic epithelium pushed above the dysplastic proliferation (**f**) There is typically a sharp line between metaplastic and dysplastic epithelium: p16+ dysplastic epithelium (**g**) and CK17 immunoreactivity (**h**)

Condylomas were consistently negative for p16 and CK17. Dysplastic lesions were divided into low-grade SIL/CIN I (*n* = 30) and high-grade SIL (CIN II, *n* = 5; CIN III *n* = 30). CIN I was negative for CK17 in all cases and negative for p16 in 27/30 cases. Three of 30 p16+ cases of CIN I were identified together with CIN III in LEEP specimens. In lesions of CIN II and CIN III, the entire dysplastic epithelium revealed strong nuclear and cytoplasmic immunoreactivity with the antibody to p16 (Figure 2a), while CK17 was uniformly negative (Figure 2b,c). Strong and uniform nuclear immunoreactivity with the antibody to p63 (Figure 2d) corresponded to the extent of dysplasia. CIN II lesions revealed near full-thickness staining with the antibody to p16 (Figure 2e), while the proliferating basal and suprabasal dysplastic cells were non-reactive with the antibody to CK17 (Figure 2f).

**Figure 3:**
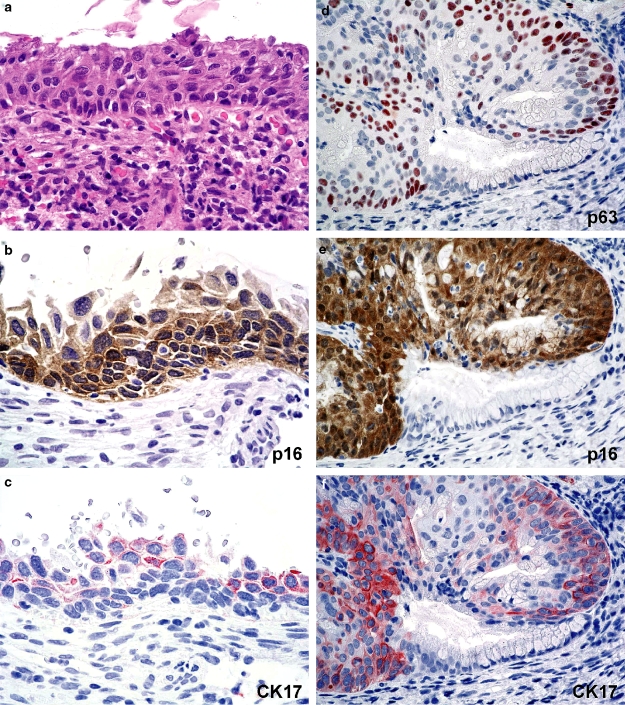
Two examples of squamous lesions signed out as atypical immature metaplasia. A cervical biopsy specimen of a 32-year-old woman (a–c) revealing a flat squamous lesion several cell layers thick with disorderly stratification and nuclear enlargement (**a**). In step sections, the dysplastic features become more prominent and p16 stains strongly positive (**b**), while cytokeratin (CK) 17 is largely negative (**c**). Another cervical biospy of a 40-year-old woman (**d**–**f**) shows a squamous lesion involving an endocervical gland. p63 highlights the nuclei of basal and suprabasal cells (**d**), while p16 (**e**) shows strong uniform positivity of the entire stratified epithelium and CK17 stains the basal proliferating cells (**f**).

Lesions classified as AIM or metaplasia with atypia (*n* = 20) at our institutions could be reclassified as chronic cervicitis with immature metaplasia (7/20) based on CK17 positivity and p16 negativity and into CIN III (10/20) based on p16 positivity and CK17 negativity. Three of 20 lesions termed AIM showed atypical squamous proliferation with significant atypia, which stained positive for both CK17 and p16 and were classified as CIN III based on the p16 staining (Figure 3a–f; Table 1).

**Table 1:** Summary of immunohistochemical staining profile, atypical immature metaplasia and cervical intraepithelial neoplasia (CIN) III

	p16	CK17	p63
Ectocervix, mature metaplasia	Negative	Negative	Basal cell nuclei
Immature metaplasia	Negative	Positive in all proliferating cells	Basal/suprabasal cell nuclei of proliferating cells
CIN III	Positive in all dysplastic cells	Negative in dysplastic cells	Nuclei of all dysplastic cells
Atypical immature metaplasia (AIM)	10/20 positive*	10/20 negative	Full-thickness positivity
	7/20 negative†	7/20 positive	Basal/suprabasal nuclei
	3/20 positive*	3/20 positive	Basal/suprabasal nuclei to full-thickness positivity

* Reclassified as CIN III.

† Reclassified as immature metaplasia.

## Discussion

Our results show that a mutually exclusive reciprocal immunohistochemical profile of CK17 and p16 allows the separation of immature metaplasia with or without reactive atypia from CIN III. Immature metaplasia was consistently characterized by strong, uniform CK17 staining of the proliferating cells with concomitant p16 negativity, while high-grade dysplasias/CIN III revealed a mirror image immunohistochemical profile with strong diffuse staining of all dysplastic proliferating cells with p16. The proliferation marker p63 showed a direct correlation between the degree of labelling and the degree of dysplasia. Atypical squamous lesions featuring both metaplastic changes with significant atypia and concomitant staining of both CK17 and p16 were classified as high-grade dysplasia (CIN III).

The cervical transformation zone displays a wide range of morphological responses to inflammation and/or HPV infection, presumably as a function of variations in target cell differentiation.^16^ High-grade cervical dysplasias have been postulated to arise through HPV infection of reserve cells. Reserve cells infected with high-risk HPV give rise to p16+ CIN, while a regenerative metaplastic process produces a CK17+ squamous epithelium. CK17 is a marker for basal cells of ‘complex’ epithelia whose expression does not correlate with HPV infection or dysplasia. In our view, CK17 expression in pseudo-stratified epithelia merely reflects a metaplastic phenotype/process. We disagree with Smedts *et al*.,^14^ who regard CK17 expression in high-grade CIN as a marker of progression to invasive cancer. The dual expression of CK17 and p16 in atypical squamous lesions with metaplastic features rather supports the hypothesis of Ma *et al*.^5^ that CIN III alternatively may develop via HPV infection of metaplastic cells.

AIM has been used as a diagnostic term in recent decades for cervical lesions with metaplastic and dysplastic features, but intra- and interobserver reproducibility has been poor. Some surgical pathologists may use this term to defer an unequivocal judgement of a cervical lesion with the unsatisfactory consequence of close clinical follow-up of the patient, since a large number of patients with this diagnosis show eventual progression to CIN III within 2 years. The term ‘AIM’ was coined in the early 1980s when immunohistochemistry in routine surgical pathology was in its infancy and reliable antibodies and markers to distinguish these two entities were not available. Since the marketing of antibodies to p16 as a surrogate marker for HPV-induced high-grade dysplasia, many cases of AIM would be reclassified as CIN III.^6^ Since p16 expression correlates well with high-risk genotype HPV infection, HPV genotyping may not produce additional information. AIM should not be used as a diagnostic term in histopathology, it should rather serve as a helpful ‘mental image’ descriptor of a worrisome lesion which needs further work-up, e.g. immunohistochemistry for p16 and/or CK17 to determine the biological significance of such a lesion.

## Abbreviations

AIM: atypical immature metaplasia
CDK: cyclin-dependent kinase
CIN: cervical intraepithelial neoplasia
CK: cytokeratin
HPV: human papillomavirus
LEEP: loop electrosurgical excision procedure
pRb: hypo-phosphorylated retinoblastoma protein
SIL: squamous intraepithelial lesion
